# Current biological approaches for management of crucifer pests

**DOI:** 10.1038/s41598-021-91088-4

**Published:** 2021-06-04

**Authors:** Saini Mayanglambam, Kabrambam Dasanta Singh, Yallappa Rajashekar

**Affiliations:** 1 Insect Bioresource Laboratory, Animal Bioresources Programme, Institute of Bioresources and Sustainable Development, Department of Biotechnology, Govt. of India, Takyelpat, Imphal, Manipur 795001 India; 2 School of Biotechnology, Kalinga Institute of Industrial Technology, Deemed To Be University, Bhubaneswar, Odisha India

**Keywords:** Zoology, Entomology, Ecology, Agroecology

## Abstract

Cabbage is considered as one of the most commonly found vegetables and it has been cultivated in large areas throughout the year. As it is mostly grown in large areas, higher rate of pest infestation likely to occur, which hinder its total production and consumption. However, continuous use of synthetic pesticides in agricultural pest management often leads to various negative impacts such as development of resistance by the pest, adverse effect on non-target organisms and hazardous effect on environment. These drawbacks led to an alternative approaches for control of crucifer pests that are cost effective, biodegradable, low toxic effect on non-target organisms and eco-friendly. This review brings together all the information of different biological practices for management of crucifer pests and list of botanical insecticides and entomopathogenic organisms that are being reported. This will help in establishing the knowledge of limited studies on pest management using different biological control methods to more challenging research and conveys the importance of pest management system for taking research forward.

## Introduction

Among the vegetables, Crucifers are important winter crop consist of cabbage, cauliflower, mustard, broccoli and radish. Cabbage, *Brassica oleracea* var. *capitata* L. is the main temperate crucifers crop that cultivates widely in different climatic regions around the world. Worldwide, India occupies the second position in the production of cabbage after China. Of the total area of vegetable grown in India, 5% is occupied by cabbage (State of Indian Agriculture, 2015–2016)^1^. Cabbage is considered as one of the most important group of vegetables and it has been cultivated in large areas throughout the years. Since cabbage is more intensively cultivated, it resulted in higher rate of pest infestation, which hinders its total production and consumption^2^. Some of the major pests of crucifers are *Pieris brassicae* L. (Lepidoptera: Pieridae)^3^, *Plutella xylostella* L. (Lepidoptera: Plutellidae)^4^, *Brevicoryne brassicae* L. (Hemiptera: Aphididae)^5^ and *Trichoplusia ni*. Hübner (Lepidoptera: Noctuidae)^6^.

Protection of vegetable crops from numerous insect pests primarily depends on the use of synthetic pesticides^122^. However, prolonged and excessive use of synthetic pesticides has led to several side-effects like development of resistance by the pest, adverse effect on non-target organisms and hazardous effects on environment. All these problems bring the sustainability of ecosystem to danger^7^. As the population of resistant pest and detrimental effects on environment rises, it requires constant support to search for an alternative control measures to reduce their spread. One promising way is to incorporate the use of biological sources such as botanical insecticides in pest management system which has resulted less negative impacts on ecosystem^8,9^.

Botanicals insecticides are chemical compound derived from plants that has the properties to kill, inhibit and repel the target pest^9,10^. These substances that are being produced naturally can be extracted and used in the formulation of commercial insecticides. Using extracts of plant material like leaves, stem, root, bark and seeds as insecticidal substances for management of crop pest has been practised for two millennia and continue the same in organic farming^11^. Some of the repellent plants can produce toxic substances and play an important role to protect against insects and pathogens^12^. This paper reviews the management of crucifer pests using current pest management strategies such as biological control practices, botanical insecticides and entomopathogenic microorganisms.

## Overview of pests of cabbage

Many insect pests hamper cabbage cultivation and the most destructive pest is *P. xylostella* which can reduce the yield of cabbage by 52% in India, if huge number of pests appeared in the field^13^. Other major insect pests on cabbage and cruciferous crops are *Crocidoloma pavonana* Fabricius (Lepidoptera:Pyralidae)^14^,, *P. brassicae*^15^, *Spodoptera litura* Fabricius (Lepidoptera: Noctuidae) and *T. ni*^16^.They infested the crucifers mostly in dry seasons and larvae start infesting the crops from their young stage and attacked the head at maturity^17^. *C. pavonana* fed on the under surface of the leaves by leaving the veins causing skeletonization of leaves. *P. xylostella* larvae initially fed on the leaves causing small holes and entirely damaged the cabbage. *T. ni* defoliates the leaves by burrowing through 3–6 layers of cabbage. *H. undalis* usually damage on outer surface of cabbage and continue feeding into the terminal bud damaging the entire cabbage plant^17,18^.

## Current biological control of Crucifer pests

### Habitat management

Habitat manipulation or management is one of the most sustainable ways of managing pests by promoting their natural enemie^19^.It involves different approaches like intercropping, push pull method and insectary plant. Intercropping can be achieved by planting secondary or tertiary crop near the main crop or by incorporating non crop plants for certain specific functions for example, providing nectar and pollen for predator and parasitoids^20^. There are many reports on effective intercropping control method such as plantation of tomato inside the cabbage plot reduced the population of many adult butterflies of *P. xylostella* and *P. rapae* as compared to the monoculture cabbage plot. It is likely due to confusing visual cues and volatiles receive from tomato which masks the cabbage. However, it was reported that there was inconsistency between the damage index and population of pest^21^. As suggested by Xu et al*.*^22 ^ decreasing pest population in intercropping plots in turn increase the pest damage index in monoculture plot. The cause of this might be due to the variation in nitrate concentration of outer layers of cabbage leaves which is higher in intercropped plot than monoculture plot. Another study concluded that, *Ocimum gratissimum* L. can reduce the population of three cabbage pest [*H. undalis, P. xylostella* and *Spodoptera littoralis* Boisd. (Lepidoptera: Noctuidae)] when grow in an alternate row with cabbage^23 ^. In another study, using of onion and tomato as an intercropped plant with cabbage as host plant could be taken as the most reasonable and inexpensive pest management strategy when compared to other methods^21^. With these studies, intercropping of certain plants like tomato, tulsi etc. with cabbage can be used preferably as an alternative for synthetic pesticides in management of cabbage pests.

### Regulating the planting period of crucifers

Regulating planting period of crucifers would be able to control certain insect infestations and can help in reducing the use of synthetic insecticides. Variables in climatic conditions play a significant role in the population of crucifer’s pest since they have a short generation time and rapid reproductive rates^24^. It also greatly depends on the temperature which may lead to an increase in infestation by rapid rises of pest population or reducing mortality of pest^25^. Impact on crop performance by planting dates is because of the changed in abiotic and biotic factors. In the cabbage field plot, the pest population started increasing from February and the highest peak occurred in April. Multiplication of pests preferred the hot climatic condition (off-season) but in cold condition (Nov-Feb) very few insects infest the cabbage^26^. According to Tanyi^27^ late plantation of cabbage (April) reduce the pest population of cabbage looper larvae, webworm larvae and *P. xylostella* when compared to normal and early plantings. This method is considered a feasible, cost-effective pest management strategy that can be implemented by the farmers. In a study, Viraktamath et al*.*^28^ reported that *P. xylostella* highly damage the leaf of cabbage planted in the first week of January in comparison with those planted in the first week of December, but the head of cabbage were not marketable in both cases. From this study, it concluded that temperature plays an important role in regulating the pest population of crucifers as hot and dry condition increases the pest population as compared to pests. Increase in temperature leads to an increase in infestation by rapid rises of the pest population.

### Push–pull strategies

**Figure 1:**
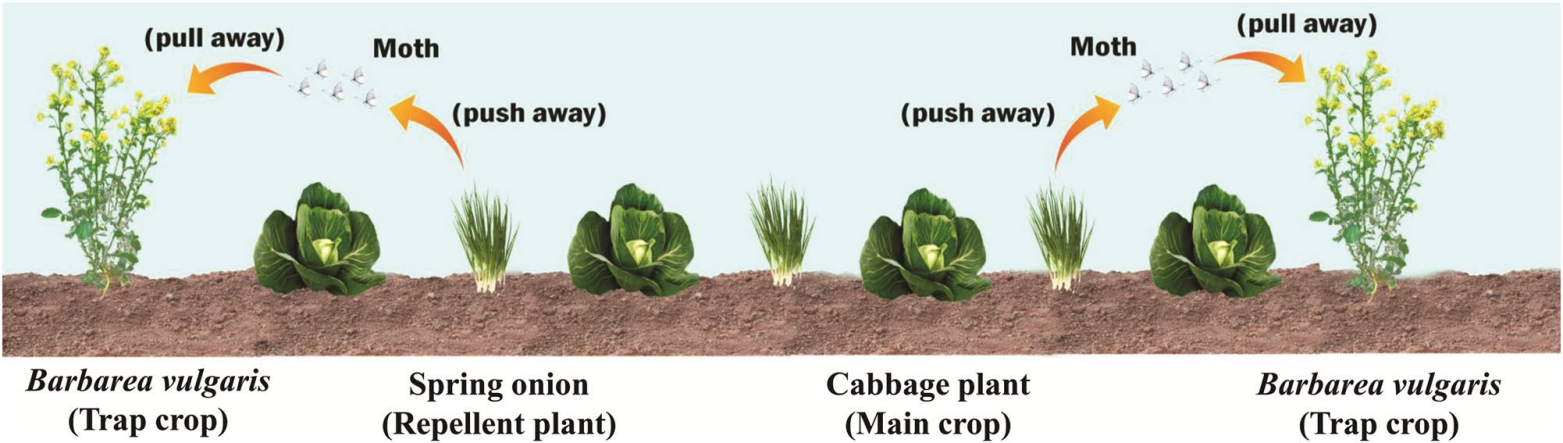
A schematic representation of the management of cabbage moth by using repellent "push" plant and trap "pull" plant. When Cabbage (maincrop) is planted with spring onion (repellent) non-host intercrop plant and simultaneously with attractive *B. vulgaris,* Yellow rocket cress (trap plant) as a barrier plant, it reduces the infestation of cabbage by cabbage moth. This occurred by repelling away the cabbage moth, that were trying to feed on the cabbage, from the push plant using stimuli that alter the host fragrance and at the same time pull away by the trap plant using highly attractive stimuli.

In push pull method, one repellent plant is planted within the crop to repel the pest and another attractant plant species is planted in the surrounding field to attract the pest^20^. The “push–pull” strategy is a technique that brings together both negative and positive impulse to repel the pests from the host plant and consequently trap the herbivores by the trap plants grows at the surrounding of host target^29^. At present, this method has been implemented approximately by 70,000 agronomist^30,31^. Presently, the most effective technique of agricultural pest management, the push–pull method, was practiced successfully and developed in Africa^32^. It required low efforts and it is an organic agricultural pest management system^33^. The techniques include both the combined use of trap crops and intercrops. The plant used as trap crops and intercrops must be suitable for the farmers and should be able to damage the natural enemies^32^. Some of the repellent plants that have been used as a push for controlling stem borers in maize are *Melnis minutiflora* P.Beauv, *Desmodium unicinatum* Jacq.DC or *Desmodium intortum* Mill., that can pull away target pests to the trap plants mainly *Pennisetum purpureum* Schumach. or *Sorghum vulgare* var *sudanense* Hitchc^34^. An example of trap plant is *Barbarea vulgaris* W.T.Aiton, which was reported and can attract the cabbage pest, *P. xylostella* but there were complications in field management practices as the plant is not suitable growing in arable fields^35^. Another case is use of onion or tomato (Fig. 1) as an intercropped plant with cabbage as host plant could be taken as the most reasonable and inexpensive pest management strategy when compared to other methods. Successful method of intercropping method using onion and tomato is probably due to the confusing volatiles and visual signals that can in return repelled the cabbage pests^21^.

### Pheromone based product for cruciferous pest management

Pheromones are a low molecular weight volatile organic molecule produced by insect to produce a behavioral response from another individual of the same species^36^. More than 1,600 pheromones and sex attractants have been reported^37^. According to Witzgall et al*.*^38^ Sex pheromones are mainly used to control the pest in an agricultural field. One of the advantages of using pheromone in pest management system is showing no adverse effects on non-target and beneficial insects as they have higher degree of specificity to one specific insect species only. Management of pest population can also be done by using synthetic pheromones where it can mask the natural pheromones produced by the lepidopteron pest and disrupt the olfactory communication of opposite sex which results in mating disruption. Mating disruption using synthetic pheromone has been considered as a feasible pest management technique^39^. However the efficacy of mating disruption is highly dependent on population density of pest as large number of pest populations are more difficult to control than less populations^40^. It has been reported that DBM sex pheromones isolated from the female moths i.e., (Z11-hexadecenal, Z11-hexadecenyl acetate in the range of 8 + 2 to 4 + 6 and addition of 1% Z11-hexadecen-1-ol were used in mass trapping of male moths in a cabbage field^41^.

## Botanicals against crucifer pests control

India is among the leading country that gains insight in developing natural botanical insecticides as most of the people still focused on indigenous traditional knowledge for controlling insect pest in the field^42^. Botanicals are natural chemical compounds derived from plants^43^.They showed different biological activities such as repellents, insecticides, fungicides and bactericides^42,44^. Some of the plants that have been reported to protect crucifer crops against insect pests are shown in (Table 1).

**Table 1:** List of some of the insecticidal plants used in management of crucifer pests.

Sl.No	Plant species (common name & Family)	Parts of the plant	Target pests	References
1	*Acorus calamus* L. (Sweet flag) Asteraceae	Leaf	*P. xylostella* Diamondback Moth & *Spodoptera frugiperda* Fall armyworm (Lepidoptera: Noctuidae)	Kumar et al*.*^74^
2	*Ageratum conyzoides* L. (White weed) Asteraceae	Leaf	*P. xylostella* & *B. brassicae* Cabbage aphid	Rioba and Stevenson^75^
3	*Alpinia galanga* L. Willd. (Siamese ginger) Zingiberaceae	Rhizomes	*S. frugiperda*	Datta et al*.*^76^
4	*Alpinia katsumadai* Hayata. (Blue ginger) Zingiberaceae	Seeds	*P. xylostella*	Hwang et al*.*^77^
5	*Annona cherimola* Mill. (Cherimoya) Annonaceae	Seeds	*S. frugiperda*	Castillo-Sánchez et al*.*^78^
6	*Annona squamosal* L. (Custard apple) Annonacea	Seeds	*P. xylostella*	Leatemia & Isman^79^
7	*Artemisia annua* (L.) (Sweet worm wood) Asteraceae	Seeds	*P. xylostella*	Okwute^80^
8	*Aspidosperma pyrifolium* Mart. & Zucc. (Pereiro) Apocynaceae	Leaf	*P. xylostella*	Torres et al*.*^81^
9	*Azadirachta indica* A Juss. (Indian lilac) Meliaceae	Leaf	*P. brassicae* Large Cabbage white	Sharma & Gupta^82^
10	*Bobgunnia madagascariensis (Desv.) (Snake bean plant) Fabaceae*	Fruit	*P. xylostella*	Mazhawidza & Mvumi^5^
11	*Bunium persicum* Boiss. (Black Jeera) Apiaceae	Fruit	*T. ni* Cabbage looper	Khanavi et al*.*^83^
12	*Cephalotaxus sinensis* (Rehder & E.H.Wilson) (Plum Yew) Cephalotaxaceae	Leaf	*P. xylostella*	Ma et al*.*^84^
13	*Clerodendrum inerme* L. (Glory bower) Lamiaceae	Leaf	*P. xylostella*	Yankanchi & Patil^85^
14	*Corymbia citriodora* Hook. (Lemon scented gum) Myrtaceae	Leaf	*P. xylostella*	Filomeno et al*.*^86^
15	*Cucurma longa* L. (Turmeric) Zingiberaceae	Rhizomes	*T. ni*	de Souza Tavares et al*.*^87^
16	*Cymbopogon citratus* (DC.) Stapf. (Lemon Grass) Poaceae	Leaf	*T. ni*	Tak and Isman^88^
17	*Cymbopogon schoenanthus* (L.) Spreng (West Indain Lemon grass) Poaceae	Leaf	*P. xylostella*	Sanda et al*.*^89^
18	*Dodonaea viscosa* (L.) Jacq (Hopseed bush) Sapindaceae	Seeds	*P. xylostella*	QIN et al*.*^90^
19	*Elettaria cardamomum* L. (Green cardamom) Zingiberaceae	Whole plants	*B. brassicae*	Jahan et al*.*^91^
20	*Eupatorium adenophorum* Spreng. (Crofton Weed) Asteraceae	Aerial part	*P. xylostella*	Adebisi et al*.*^92^
21	*E.adenophorum* Spreng. *& Lantana camara* L. (Lantana) Verbenaceae	Aerial parts	*P. brassicae*	Khan et al*.*^93^
22	*Apium nodiflorum* L.Lag. (Fools Water Cress) Apiaceae	Aerial parts	*T. ni*	Afshar et al*.*^94^
23	*Jatropha gossypifolia* L. (Cotton leaf) Euphorbiaceae	Leaf	*S. frugiperda*	Bullangpoti et al.^95^
24	*L.camara* L	Leaf	*B. brassicae*	Mvumi & Maunga^96^
25	*Maerua edulis* (Gilg & Gilg-Ben.) DeWolf. (Blue bush cherry) Capparaceae	Leaf	*P. xylostella*	Mazhawidza & Mvumi^5^
26	*Melia azedarach* L. (Chinaberry tree) Meliaceae	Leaf	*P. xylostella*	Kumar et al*.*^97^
27	*Melia volkensii* Gurke. (Melia) Meliaceae	Seeds	*T. ni*	Akhtar et al*.*^98^
28	*M.volkensii* Gurke	Seeds	*P. xylostella* &*T. ni*	Akhtar & Isman^99^
29	*Muntingia calabura* L. (Panama berry) Muntingiaceae	Fruits and flowers	*P. xylostella*	Bandeira et al*.*^100^
30	*Origanum vulgare *L. (Oregano) Lamiaceae	Aerial parts	*P. xylostella*	Nasr et al*.*^101^
31	*Otostegia persica* Boiss. (Tinjut) *Lamiaceae*& *Peganum harmala* L. (Wild Rue) Zygophyllacea	Seeds	*B. brassicae*	Shafiei et al.^102^
32	*Oxandra xylopioides* Diels. Annonaceae	Leaf	*S. frugiperda*	Castillo-Sánchez et al*.*^78^
33	*Panax ginseng C.A.MEYER* (Chinese ginseng) Araliaceae	Leaf and Stem	*P. xylostella*	Yang et al*.*^4^
34	*Pharbitis purpurea* L. (Morning glory) Convolvulacea	Seed kernels	*P. xylostella*	Xu et al*.*^103^
35	*Ricinus communis* L. (Castor bean) Euphorbiaceae	Seed kernels	*P. xylostella*	Kodjo et al.^104^
36	*Rosmarinus officinalis* L. (Rosemary) Lamiaceaea	Aerial parts	*T. ni*	Tak et al*.*^61^
37	*Satureja hotensis* L. *(Summer savory Meliaceae *&*Cuminum cyminum* L. (Cumin) Apiaceae	Leaf	*P. brassicae*	Khorrami et al*.*^105^
38	*Vitex negundo* (L.) (Chinese Chase tree) Lamiaceae	Leaf	*P. xylostella*	Yankanchi & Patil^106^

Botanical insecticides served as effective and safer alternatives of synthetic insecticides, as they are readily available and safer for the non-target organisms and for the environment^45,46^. Some common chemical compounds reported from plants are Pyrethrins, Nicotine, Rotenone, Azadirachtin, Limonene, Limone, Linalool, Citronellal, Artemisinin, Diterpene, Coumarins, Annonin^47,48^. According to 2012 report, Ministry of agriculture approved nine botanicals insecticides along with garlic and neem extracts^49^. Those seven botanical insecticides include *Cymbopogon * spp. Spreng., *Sophora * spp. L., *Annona squamosa* L., *Tripterygium wilfordii* Hook.F., *Apocynum venetum* L., *Eucalyptus globulus* Labil. and *Milletia pinnata* L. They have been commercialized by Ministry of Agriculture^50^. Studies have reported that azadirachtin from Neem, *Azadirachta indica* A.juss and lantanine from *Lantana camara* L. exhibit defensive mechanism against insect’s pests. Azadirachtin is considered as one of the most effective botanical insecticide and helped in management of many agricultural pests^51,52^. As reported by Shah, F. M. et al^122^ botanically derived commercial formulation NeemAzal was just as effective as synthetic insecticides in terms of pest suppression and marketable yield. Some of the insecticidal plant used in management of pests in cabbage and cauliflower are leaf extract of *Melia azedarach* L.^53^*, Tagetes minuta* L.*, Cymbopogon flexuosus* Nees ex Steud*, Acorus calamus* L*., Eupatorium adenophorum* Spreng and *Artemisia maritima* L.^54^*.* Although some agricultural organizations often recommended using botanical insecticides over synthetic pesticides there are some drawbacks like having poor scientific evidence on the efficacy and safety of botanical insecticides^55^. One of the factors that control the efficacy of the botanical insecticides mainly depends on concentration of active constituents and its varying contents^56^. Variable concentration of active constituents mainly resulted from the varying concentration of secondary metabolite contents which is caused by an extensive factor like the genotype of plants, different environmental factors and plant developmental stage^57^. Besides the above factor, an important factor could be due to the storage condition as the active constituents present in botanical insecticides may deteriorate gradually while storing^58^. Some other factors like a method of application of bioactive compound and a structural membrane of the target pest and its body conformation is responsible for altering the bioactivity of compounds and its toxicity^59^. It has been reported that the synergistic activity of plant essential oil constituents, may enhance the penetration effect into the insect integument. In a study of constituents of rosemary essential oil i.e., 1, 8-Cineole and camphor against *T. ni*, it was found out that mixture of 1, 8-Cineole and camphor oil gave higher toxicity than the one applied individually on *T. ni*^60^. In another study, positive synergistic effects between the constituents of lemon grass oil was shown greater insecticidal activity against the *T.ni* although some minor constituent like limonene were less effective than citral the main active compound^61^ and it was also reported that the combination of three major components (thymol, p-cymene and linalool) of thyme oil which were obtained from *Thymus vulgaris* L. (Thyme) the binary mixtures have shown synergistic activity against the third instar larvae of *S. litoralis* ^62^.

## Microbial control agent against crucifer pest

Microbial biopesticides are products developed from microorganisms like bacteria, fungi, nematode and viruses or its products that are used to control the agricultural pest and also play an important role as an alternative tool to chemical pesticides for their eco-friendly nature^63^. According to NBAIR 2017 report, minimum of 15 biopesticides based on microbes have been developed in India with 970 commercial formulations registered^64^. Some of the microbial control agents against crop pests are discussed here in Table 2.

**Table 2:** List of some of the entomopathogenic microbes used in management of crucifer pests.

Sl.No	Entomopathogenic microbes	Target pest	Types of microbes	References
1	*Bacillus thuringinesis* Berliner *ssp* kurstaki	*T. ni*	Bacterium	Ramanujam et al*.*^107^
2	*Bacillus thuringinesis* var. galleriae	*Helicoverpa armigera* HübnerCotton Bollworm (Lepidoptera:Noctuidae) & *P. xylostella*	Bacterium	Singh et al*.*^108^
3	*Beauveria bassiana –* Myco Jaal	*P. xylostella*. & *P. brassicae*	Fungus	Ghosh et al*.*^109^ Srinivasan et al*.*^110^ & Singh et al*.*^111^
4	*Beauveria brongniartii*	*S. litura*	Fungus	Lin et al*.*^112^
5	*Cabbage looper* (TrniSNPV)	*T. ni*	Virus	Singh et al*.*^108^
6	*Chromobacterium subtsugae*	*P. xylostella*	Bacterium	Martin et al*.*^113^
7	*Diamond back moth GV* (PlxyGV)	*P. xylostella*	Virus	Singh et al*.* ^108^
8	*Egyptian cotton leafworm NPV* (SpliNPV)	*S. littoralis*	Virus	Singh et al*.*^108^
9	Granulosis Virus	*P. rapae*	Virus	Ramanujam et al*.*^107^
10	*Heterorhabditis bacteriophora*	*P. xylostella*& *P. brassicae*	Nematode	Rodriguez et al*.*^114^ & Abbas et al*.* ^115^
11	*Isaria fumosoroseus*	*P. xylostella*	Fungus	Huang et al*.*^116^
12	*Nomuraea rileyi*	*S. litura*	Fungus	Lin et al*.*^112^
13	Nuclear Polyhedrosis Virus	*Mamestra brassicae L. Cabbage moth* (Lepidoptera:Noctuidae)	Virus	Kunimi^117^
14	*Photorhabdus luminescens*	*P. brassicae*	Nematode	Mohan et al*.*^118^
15	*Steinernema carpocapsae*	*P. xylostella*	Nematode	Baur et al*.*^119^ & Sunanda et al*.*^120^
16	*Steinernema glaseri*	*P. brassicae*	Nematode	Abbas et al*.*^114^
17	*Xenorhabdus nematophila*	*P. xylostella*	Nematode	Razek et al*.*^121^

Fungi species which are pathogenic to insect pests are called entomopathogenic fungi. The most commonly used entomopathogenic fungi are *Beauveria bassiana (Balsamo)Vuillemin, B. brongniartii (Sacc.)Petch, Metarhizium anisopliae (Metschn.)Sorokin, Lecanicillium lecnii (Zimmerman) Gams & Zare, Hirsutella thompsonii Fisher, Cladossporium oxysporium Berk & M.A.Curtis)* and *Isaria fumosorosea (Wize)*^65,66^*.* Based on the report of entomopathogenic bacteria, the most commercially used microbial pesticide belongs to gram positive bacteria mostly in the genera of *Bacillus*,* Paenibacillus* and *Lysinibacillus*^67^. More than 30 products developed from the sub species *kurstaki* of *B.thuringiensis* are effective against bollworms, loopers and other lepidopterans and also two viruses namely Helicoperva armigera nucleopolyhedrovirus and Spodoptera litura mucleopolyhedrovirus were registered to control two lepidopteran pests i.e., *Helicoverpa* spp., *S. litura* and *S. exigua*^53^.

Although microbial pesticides have many advantages for control of crucifer pest, several factors limit the commercial production, and their efficacy also varies among the stage of larvae, strains, environmental condition and target pests. The efficacy of these products is highly effective when applied to the young larvae (first and second instars larva) and reapplication when insect population increases^68–70^. Some of the factors that limit the commercialization of microbial pesticides include low microbial counts, as rapid production of entomophthoralean fungi species is quite low due to difficulty in development of conidia and its short-lived which makes impossible in creating a period of vast applications. For this one should try to increase the production of resting spores and competent mycelia of entomophthoralean species by developing effective methods which will ultimately increase the efficacy of these fungi^71^. Another factor is the shelf life of entomopathogenic microbes, where storage facilities are not yet developed in rural areas^72^. Poor solubility of the some of the formulations in water is also one of the challenges^73^. Despite of all the challenges, several methods need to be followed like enhancing the microbial production and formulation, learning the proper idea of microbial pesticides being incorporated into integrated systems and their relations with the external environment, accepting the advantages like efficacy, safety etc. while comparing with synthetic pesticides and approved^71^.

## Conclusions

As biological control of pest can be an alternative to synthetic pesticides, effectiveness and maintenance of developing control method for crucifer pests must be considered. Some of the criteria that should be encountered for developing a proper biological control methods are (1) adopting proper guidelines to the farmers about various approaches of pest management in a comprehensive manner, (2) providing awareness programme for the negative impacts of used of synthetic pesticides for better cooperation of the farmers (3) having proper taxonomical knowledge on insectary plants, trap crops and insecticidal plants and (4) maintained authentic research data during laboratory practices to be commercialised later. These approaches can provide the importance of the economic benefits of using biological control method over synthetic products and will gain insight of accepting the sustainable way of crucifer pest management. The ultimate challenge will be to adopt the use of biological pest management technologies in a cost effective manner so that farmer can easily access those approaches.
