# Author Correction: Current biological approaches for management of crucifer pests

**DOI:** 10.1038/s41598-021-98185-4

**Published:** 2021-09-28

**Authors:** Saini Mayanglambam, Kabrambam Dasanta Singh, Yallappa Rajashekar

**Affiliations:** 1 Insect Bioresource Laboratory, Animal Bioresources Programme, Institute of Bioresources and Sustainable Development, Department of Biotechnology, Govt. of India, Takyelpat, Imphal, Manipur 795001 India; 2 School of Biotechnology, Kalinga Institute of Industrial Technology, Deemed To Be University, Bhubaneswar, Odisha India

Correction to: *Scientific Reports* 10.1038/s41598-021-91088-4, published online 04 June 2021

The original version of this Article contained errors.

Reference 122 was omitted, and is listed below.

Shah, F. M. *et al.* Field evaluation of synthetic and neem-derived alternative insecticides in developing action thresholds against cauliflower pests. *Sci. Rep.* **9**(1), 1-13 (2019).

Consequently, the text in the Introduction,

“Protection of vegetable crops from numerous insect pests primarily depends on the use of synthetic pesticides”

now reads:

“Protection of vegetable crops from numerous insect pests primarily depends on the use of synthetic pesticides^122^”

Under the same section,

“Some of the major defoliating caterpillars that cause significant damage to the crucifers are *Pieris brassicae* L. (Lepidoptera: Pieridae)^3^, *Plutella xylostella* L. (Lepidoptera: Plutellidae)^4^, *Brevicoryne brassicae* L. (Hemiptera: Aphididae)^5^ and *Trichoplusia ni.* Hübner (Lepidoptera: Noctuidae)^6^”

now reads:

“Some of the major pests of crucifers are *Pieris brassicae* L. (Lepidoptera: Pieridae)^3^, *Plutella xylostella* L. (Lepidoptera: Plutellidae)^4^, *Brevicoryne brassicae* L. (Hemiptera: Aphididae)^5^ and *Trichoplusia ni.* Hübner (Lepidoptera: Noctuidae)^6^”

And under the subheading ‘Overview of pests of cabbage’, the text,

“Many insect pests hamper cabbage cultivation and the most destructive pest is *P*. *xylostella* which can reduce the yield of cabbage by 80% if huge number of pests appeared in the field^13^”

now reads:

“Many insect pests hamper cabbage cultivation and the most destructive pest is *P. xylostella* which can reduce the yield of cabbage by 52% in India if huge number of pests appeared in the field^13^”

And the text under the subheading, ‘Botanicals against crucifer pests control’,

“Studies have reported that azadirachtin from Neem, *Azadirachta indica* A.juss and lantanine from *Lantana camara* L. exhibit defensive mechanism against insect’s pests. Azadiractin is considered as one of the most effective botanical insecticide and helped in management of many agricultural pests^51,52^. Some of the insecticidal plant used in management of pests in cabbage and cauliflower are leaf extract of *Melia azedarach L.*^53^, *Tagetes minuta* L., *Cymbopogon fexuosus* Nees ex Steud, *Acorus calamus* L., *Eupatorium adenophorum* Spreng and *Artemisia maritima* L.^54^.”

now reads:

“Studies have reported that azadirachtin from Neem, *Azadirachta indica* A.juss and lantanine from *Lantana camara* L. exhibit defensive mechanism against insect’s pests. Azadirachtin is considered as one of the most effective botanical insecticide and helped in management of many agricultural pests^51,52^. As reported by Shah, F. M. et al.^122^ botanically derived commercial formulation NeemAzal was just as effective as synthetic insecticides in terms of pest suppression and marketable yield. Some of the insecticidal plant used in management of pests in cabbage and cauliflower are leaf extract of *Melia azedarach L.*^53^, *Tagetes minuta* L., *Cymbopogon fexuosus* Nees ex Steud, *Acorus calamus* L., *Eupatorium adenophorum* Spreng and *Artemisia maritima* L.^54^.”

In addition, Reference 13 and 17 were incorrect. Therefore, in the Reference list, the text,

“13. Furlong, M. J., Wright, D. J. & Dosdall, L. M. Diamondback moth ecology and management: problems, progress, and prospects. *Annu. Rev. Entomol.* **58,** 517–541 (2013).”

now reads:

“13. Krishnamoorthy, A. Biological control of diamondback moth Plutella xylostella (L.), an Indian scenario with reference to past and future strategies in *Proceedings of the International Symposium* (eds. Kirk, A. A. & Bordat, D.) 204–211 (Montpellier, France: CIRAD 2004).”

And the text,

“17. Lim, G. S., Sivapragasam, A., & Loke, W. H. Crucifer insect pest problems: trends, issues and management strategies. In *The Management of diamondback and other crucifer pests.* Proceedings of the third international workshop, Kuala Lumpur, Malaysia. (1996).”

now reads:

“17. Mochiah, M. B., Baidoo, P. K. & Owusu-Akyaw, M. Influence of different nutrient applications on insect populations and damage to cabbage. *J. Appl. Biosci.* **38**, 2564–2572 (2011).”

There were a number of minor grammatical errors which have been amended.

The original Article has been corrected.

